# The global burden of scabies: a cross-sectional analysis from the Global Burden of Disease Study 2015

**DOI:** 10.1016/S1473-3099(17)30483-8

**Published:** 2017-12

**Authors:** Chante Karimkhani, Danny V Colombara, Aaron M Drucker, Scott A Norton, Roderick Hay, Daniel Engelman, Andrew Steer, Margot Whitfeld, Mohsen Naghavi, Robert P Dellavalle

**Affiliations:** a Department of Dermatology, University of Colorado Anschutz Medical Campus, Aurora, CO, USA; b Institute for Health Metrics and Evaluation, University of Washington, Seattle, WA, USA; c Department of Dermatology, Brown University, Providence, RI, USA; d Department of Dermatology, Children's National Medical Center, NW Washington DC, USA; e Department of Dermatology, King's College Hospital, Denmark Hill, London, UK; f Centre for International Child Health and Murdoch Children's Research Institute, University of Melbourne, Royal Children's Hospital, Melbourne, VIC, Australia; g Department of Dermatology, St Vincent's Hospital, Darlinghurst, Sydney, NSW, Australia; h Department of Epidemiology, Colorado School of Public Health, Aurora, CO, USA; i Dermatology Service, US Department of Veterans Affairs, Eastern Colorado Health System, Denver, CO, USA

## Abstract

**Background:**

Numerous population-based studies have documented high prevalence of scabies in overcrowded settings, particularly among children and in tropical regions. We provide an estimate of the global burden of scabies using data from the Global Burden of Disease (GBD) Study 2015.

**Methods:**

We identified scabies epidemiological data sources from an extensive literature search and hospital insurance data and analysed data sources with a Bayesian meta-regression modelling tool, DisMod-MR 2·1, to yield prevalence estimates. We combined prevalence estimates with a disability weight, measuring disfigurement, itch, and pain caused by scabies, to produce years lived with disability (YLDs). With an assumed zero mortality from scabies, YLDs were equivalent to disability-adjusted life-years (DALYs). We estimated DALYs for 195 countries divided into 21 world regions, in both sexes and 20 age groups, between 1990 and 2015.

**Findings:**

Scabies was responsible for 0·21% of DALYs from all conditions studied by GBD 2015 worldwide. The world regions of east Asia (age-standardised DALYs 136·32), southeast Asia (134·57), Oceania (120·34), tropical Latin America (99·94), and south Asia (69·41) had the greatest burden of DALYs from scabies. Mean percent change of DALY rate from 1990 to 2015 was less than 8% in all world regions, except North America, which had a 23·9% increase. The five individual countries with greatest scabies burden were Indonesia (age-standardised DALYs 153·86), China (138·25), Timor-Leste (136·67), Vanuatu (131·59), and Fiji (130·91). The largest standard deviations of age-standardised DALYs between the 20 age groups were observed in southeast Asia (60·1), Oceania (58·3), and east Asia (56·5), with the greatest DALY burdens in children, adolescents, and the elderly.

**Interpretation:**

The burden of scabies is greater in tropical regions, especially in children, adolescents, and elderly people. As a worldwide epidemiological assessment, GBD 2015 provides broad and frequently updated measures of scabies burden in terms of skin effects. These global data might help guide research protocols and prioritisation efforts and focus scabies treatment and control measures.

**Funding:**

Bill & Melinda Gates Foundation.

## Introduction

Scabies is a skin infestation caused by the mite *Sarcoptes scabiei* that causes a pruritic skin eruption.^1^ Given that scabies transmission occurs with person-to-person contact, scabies is particularly prevalent in resource-poor conditions and among children, and is associated with insufficient access to health-care subsidies. Scabies can occur in any setting but over the past century has become less prevalent in temperate regions and is more common in tropical, humid regions. The predominant symptom of scabies infestation is pruritus, which can be debilitating. Disruption of the skin's protective barrier function promotes secondary bacterial infections, which can lead to additional, potentially life-threatening, complications.^2^

Scabies has high prevalence in the tropics and large cumulative morbidity. Recognition of scabies on the global health agenda would increase awareness, education, and research into diagnosis, treatment, and prevention.^3^ In recognition, WHO recently formally designated scabies as a neglected tropical disease.^4^ Previous investigations have reported on scabies prevalence in specific, often low-resource, communities.^5^, ^6^, ^7^, ^8^ A systematic review of 48 population-based studies found the highest prevalence of scabies in Papua New Guinea, Panama, and Fiji.^9^ However, beyond prevalence, the extent to which scabies affects these communities is unknown. In this paper, we provide estimates for the global burden of scabies skin disease using data from the Global Burden of Disease (GBD) study.

GBD provides a way to measure and compare health loss from disease and injury across age, sex, location, and time.^10^ GBD is based on formal, systematic, and statistically rigorous analyses of effects of disease and injuries on the health of populations. As an international collaboration of more than 500 experts representing 30 countries, GBD 2015, the third iteration of the GBD process, quantified the effects of 315 diseases and injuries, including scabies, in 195 countries from 1990 to 2015.^11^ Disease burden is measured using the disability-adjusted life-years (DALYs) metric, which uniquely combines mortality (estimated using years of life lost [YLL]) and morbidity (estimated using years lived with disability [YLD]) components. By assessing disease epidemiology on a global scale, GBD has the potential to inform health policy and identify previously undervalued or neglected conditions, such as scabies. The DALY metric has broad clinical and research priority-setting implications because it assesses both the prevalence and impact of a disease and allows for comparison of various diseases. This report presents GBD 2015 results on the global burden of scabies.

**Research in context**

**Evidence before this study**

We searched PubMed and Google Scholar databases on July 15, 2017, for articles in English, Spanish, and French published before June 30, 2017, using the key word “scabies” in the title or abstract. Studies reported high-risk population-based national and subnational estimates of scabies prevalence as well as associated comorbidities such as psychological disorders. However, previous attempts to estimate the global burden of scabies skin infection were not available. The Global Burden of Disease (GBD) Study 2015 assesses scabies epidemiological data sources from a PubMed and Google Scholar literature search in English and Spanish between 1980 and 2014. Prevalence and incidence metrics were extracted from included sources and analysed with a Bayesian meta-regression modelling tool. Burden of disease is estimated as disability-adjusted life-years (DALYs).

**Added value of this study**

This study is the first global effort to measure the burden of scabies. The greatest DALYs from scabies are in tropical regions in east Asia, southeast Asia, Oceania, tropical Latin America, and south Asia, especially in children, adolescents, and the elderly.

**Implications of all the available evidence**

Increased global awareness of the burden from scabies will promote international efforts for control of this preventable disease. GBD provides high-quality estimates, which can be used to set research priorities, promote discussion, and ultimately, enact change, at local, national, and global stages.

## Methods

### Data collection

Although details of GBD methods are extensively published elsewhere,^11^, ^12^, ^13^ a brief overview specific to scabies is presented here. The GBD category of scabies is defined by the International Classification of Diseases (ICD)-9 code 133 and ICD-10 code B86. A systematic literature search was done and results were screened by title and abstract to identify relevant studies, which then underwent full-text screening and data extraction. Studies published between 1980–2014 that provided data on scabies incidence or prevalence, used samples representative of the general population that were larger than 100, and provided sufficient information on methods to assess study quality as well as rules for extracting uncertainty (standard error and 95% CI) were included. Additionally, US health insurance claims data from 2000, 2010, and 2012 were included (appendix). 38 studies on scabies prevalence in 84 countries and three studies on scabies incidence in five countries were included. All extracted scabies incidence and prevalence datapoints were age-sex split and adjusted from primary code to all code based on the claims data. These datapoints were then input into DisMod-MR 2.1, a Bayesian meta-regression tool, which estimates scabies prevalence by location, year, age, and sex. For the DisMod-MR 2.1 analysis, scabies was modelled with remission set between 1 and 9, corresponding to durations of 6 weeks to 1 year, and mortality was assumed to be zero, on the basis of available epidemiological data, expert opinion, and previous GBD studies. As a proxy for low levels of development, improved water source (proportion of population with access to sufficient quantities of water) was used as a country-level covariate. For countries or regions with missing data, DisMod-MR 2.1 uses data in nearby countries, regions, and predictive covariates to estimate data.

GBD divides disease prevalence into varying severity levels. Scabies prevalence was categorised as one severity level: disfigurement level 1 with itch or pain. This severity level corresponded to the lay description: “The individual has a slight visible physical deformity that is sometimes sore or itchy. Observers notice the deformity, which causes some worry and discomfort to the patient”. The severity level was assigned on the basis of recommendations from the GBD 2010 Skin Conditions Expert Group.^14^ The severity prevalence estimates were multiplied with a disability weight to generate YLDs for each age-sex-country-year group. Disability weights, which range from 0 (least disabling) to 1 (most disabling), assessed the degree of disfigurement with itch or pain from scabies in four population-based European surveys and an open-access web-based survey of more than 60 890 respondents.^15^ The disability weight assigned to scabies was 0·027 (95% CI 0·015–0·042). Notably, this weighting only takes into account the effect of scabies on the skin.

With scabies, YLL is assumed to be zero; YLDs were equivalent to DALYs. DALY metrics are computed as age-standardised and age-specific DALY rate per 100 000 persons and mean percent change in age-standardised DALY rate from 1990–2015. Scabies estimates are made for both sexes, 20 age groups (ranging from 0 days to >80 years), and 21 world regions that include 195 countries and territories (panel). Age-standardisation was based on GBD 2013 estimates of the standard population structure from 2010–35 based on the most recent World Population Prospects publication by the UN Population Division.^16^ To assess variance of DALYs by age for a particular region, standard deviation in each of the 20 age groups was calculated. DALY metrics were organised and analysed in Microsoft Excel, version 14.7.1. The Global Burden of Disease Study is approved by the international review board of the University of Washington until March 25, 2018.

Panel **Global Burden of Disease regions and countries within each region**

**East Asia**

China, North Korea, and Taiwan (province of China)

**Oceania**

American Samoa, Federated States of Micronesia, Fiji, Guam, Marshall Islands, Northern Mariana Islands, Papua New Guinea, Samoa, Solomon Islands, Tonga, and Vanuatu

**Southeast Asia**

Cambodia, Indonesia, Laos, Malaysia, Maldives, Mauritius, Myanmar, Philippines, Sri Lanka, Seychelles, Thailand, Timor-Leste, and Vietnam

**South Asia**

Bangladesh, Bhutan, India, Nepal, and Pakistan

**Central Asia**

Armenia, Azerbaijan, Georgia, Kazakhstan, Kyrgyzstan, Mongolia, Tajikistan, Turkmenistan, and Uzbekistan

**Central Europe**

Albania, Bosnia and Herzegovina, Bulgaria, Croatia, Czech Republic, Hungary, Macedonia, Poland, Romania, Serbia, Slovakia, and Slovenia

**Eastern Europe**

Belarus, Estonia, Latvia, Lithuania, Moldova, Russia, and Ukraine

**North Africa and Middle East**

Afghanistan, Algeria, Bahrain, Egypt, Iran, Iraq, Jordan, Kuwait, Lebanon, Libya, Morocco, Palestine, Oman, Qatar, Saudi Arabia, Sudan, Syria, Tunisia, Turkey, United Arab Emirates, and Yemen

**Western sub-Saharan Africa**

Benin, Burkina Faso, Cameroon, Cape Verde, Chad, Côte d'Ivoire, The Gambia, Ghana, Guinea, Guinea-Bissau, Liberia, Mali, Mauritania, Niger, Nigeria, São Tomé and Príncipe, Senegal, Sierra Leone, and Togo

**Southern sub-Saharan Africa**

Botswana, Lesotho, Namibia, South Africa, Swaziland, and Zimbabwe

**Eastern sub-Saharan Africa**

Burundi, Comoros, Djibouti, Eritrea, Ethiopia, Kenya, Madagascar, Malawi, Mozambique, Rwanda, Somalia, South Sudan, Tanzania, Uganda, and Zambia

**Central sub-Saharan Africa**

Angola, Central African Republic, Congo (Brazzaville), Democratic Republic of the Congo, Equatorial Guinea, and Gabon

**Tropical Latin America**

Brazil and Paraguay

**Andean Latin America**

Bolivia, Ecuador, and Peru

**Central Latin America**

Colombia, Costa Rica, El Salvador, Guatemala, Honduras, Mexico, Nicaragua, Panama, and Venezuela

**Caribbean**

Antigua and Barbuda, The Bahamas, Barbados, Belize, Bermuda, Cuba, Dominica, Dominican Republic, Grenada, Guyana, Haiti, Jamaica, Puerto Rico, Saint Lucia, Saint Vincent and the Grenadines, Suriname, Trinidad and Tobago, and Virgin Islands

**Western Europe**

Andorra, Austria, Belgium, Cyprus, Denmark, Finland, France, Germany, Greece, Iceland, Ireland, Israel, Italy, Luxembourg, Malta, Netherlands, Norway, Portugal, Spain, Sweden, Switzerland, and UK

**Southern Latin America**

Argentina, Chile, and Uruguay

**North America**

Canada, Greenland, and USA

**Asia Pacific**

Brunei, Japan, Singapore, and South Korea

**Australasia**

Australia and New Zealand

### Role of the funding source

The funder of the study had no role in study design, data collection, data analysis, data interpretation, or writing of the report. The corresponding author had full access to all the data in the study and had final responsibility for the decision to submit for publication.

## Results

The GBD 2015 global prevalence of scabies in both sexes was 204 151 715 (95% CI 177 533 726–237 466 220).^13^ Scabies caused 0·21% of DALYs from all conditions studied by GBD 2015 globally. Global age-standardised DALYs per 100 000 people from scabies was 71·11 (95% CI 39·77–116·03) for both sexes,^11^ and 70·58 (39·79–114·37) for men and 71·72 (39·90–117·83) for women (data not shown). Of 246 conditions comparatively ranked by GBD 2015, scabies ranked 101 in age-standardised global DALYs, after adverse effects of medical treatment (ranked 98), viral skin diseases (99), and *Haemophilus influenzae* type b meningitis (100), and before atrial fibrillation or flutter (102), acute lymphoid leukaemia (103), and other transport injuries (104). Global age-standardised DALYs in 20 age groups are shown in table 1.

**Table 1:** Global DALYs from scabies skin infection by age

	**Global DALYs per 100 000 people (95% CI)**
0–6 days	2·35 (1·20–4·00)
7–27 days	10·97 (5·56–18·81)
28–364 days	73·72 (37·33–125·03)
1–4 years	116·30 (59·28–205·71)
5–9 years	99·25 (48·95–183·04)
10–14 years	95·22 (46·24–164·65)
15–19 years	101·88 (50·78–177·68)
20–24 years	96·76 (46·15–176·88)
25–29 years	73·28 (35·03–141·91)
30–34 years	53·20 (26·13–96·12)
35–39 years	47·66 (23·14–86·20)
40–44 years	50·24 (23·54–89·20)
45–49 years	49·94 (24·52–92·29)
50–54 years	44·63 (21·98–83·38)
55–59 years	39·94 (19·31–69·18)
60–64 years	38·80 (19·51–67·56)
65–69 years	40·02 (20·31–71·07)
70–74 years	42·80 (21·40–76·67)
75–79 years	52·34 (25·26–92·46)
≥80 years	46·38 (24·03–78·30)

DALYs=disability-adjusted life-years.

**Figure 1:**
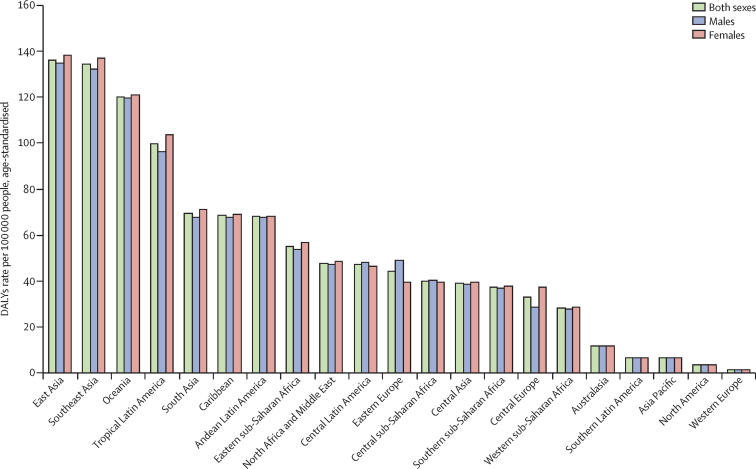
Global 2015 scabies age-standardised DALYs per 100 000 people in males, females, and both sexes DALYs=disability-adjusted life-years.

**Figure 2:**
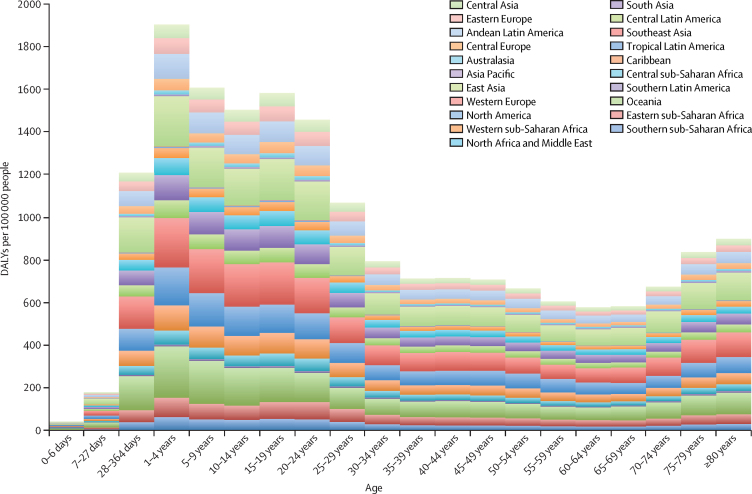
Scabies DALYs per 100 000 people by age and world region DALYs=disability-adjusted life-years.

The five world regions with the greatest age-standardised DALY burdens caused by scabies in decreasing order were east Asia 136·32 (95% CI 75·83–222·35), southeast Asia 134·57 (74·62–223·64), Oceania 120·34 (68·10–194·84), tropical Latin America 99·94 (56·75–163·50), and south Asia 69·41 (39·73–112·65; figure 1). Mean percent changes in age-standardised DALYs from 1990 to 2015 range from 21·87% (97·5% uncertainty interval [UI] 10·83 to 37·36) in North America to −7·92% (−10·07 to −5·51) in eastern sub-Saharan Africa (table 2). The regions with greatest standard deviation of DALY burdens of the 20 age groups were southeast Asia 60·1, Oceania 58·3, and east Asia 56·5 (figure 2). The regions with the lowest standard deviation of DALY burden of the 20 age groups were Asia Pacific 2·2, North America 1·8, and western Europe 0·6.

**Table 2:** Mean percent change in age-standardised DALYs

	**Mean percent change in DALYs 1990–2015 (97·5% UI)**
Global	−5·38 (−7·81 to −3·14)
North America	23·87 (10·83 to 37·36)
Southern Latin America	0·69 (−5·38 to −6·99)
Western Europe	0·32 (−4·57 to 5·46)
Australasia	0·02 (−4·97 to 5·22)
Central Europe	0·06 (−2·64 to 2·32)
Asia Pacific	−0·29 (−4·21 to 4·22)
Eastern Europe	−0·83 (−4·78 to 3·83)
Caribbean	−0·84 (−3·75 to 1·80)
South Asia	−1·12 (−5·23 to 3·17)
Central Latin America	−1·30 (−3·83 to 2·33)
Andean Latin America	−1·51 (−5·34 to 2·54)
Southern sub-Saharan Africa	−1·54 (−5·58 to 2·75)
Central Asia	−1·79 (−4·69 to 1·00)
Tropical Latin America	−2·55 (−7·21 to 2·26)
Central sub-Saharan Africa	−2·62 (−6·89 to 2·19)
Southeast Asia	−2·66 (−6·15 to 1·02)
North Africa and Middle East	−2·86 (−5·17 to −0·09)
East Asia	−3·18 (−7·71 to 1·76)
Western sub-Saharan Africa	−7·16 (−10·25 to −3·45)
Oceania	−7·12 (−10·80 to −3·04)
Eastern sub-Saharan Africa	−7·92 (−10·07 to −5·51)

Data are for both sexes from 1990 to 2015 globally and by world region. UI=uncertainty interval. DALYs=disability-adjusted life-years.

**Figure 3:**
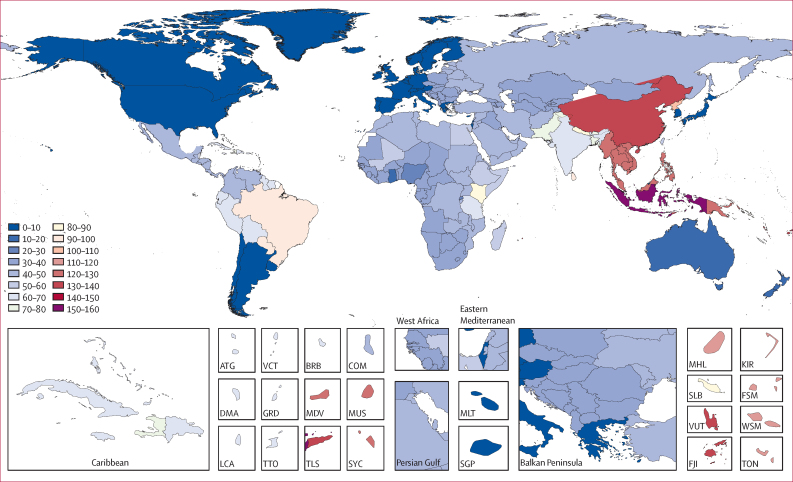
World map of scabies age-standardised disability-adjusted life-years per 100 000 people ATG=Antigua and Barbuda. BRB=Barbados. COM=Comoros. DMA=Dominica. FJI=Fiji. FSM=Federated States of Micronesia. GRD=Grenada. KIR=Kiribati. LCA=Saint Lucia. MDV=Maldives. MHL=Marshall Islands. MLT=Malta. MUS=Mauritius. SGP=Singapore. SLB=Solomon Islands. SYC=Seychelles. TLS=Timor-Leste. TON=Tonga. TTO=Trinidad and Tobago. VCT=Saint Vincent and the Grenadines. VUT=Vanuatu. WSM=Samoa.

Of the 195 countries analysed, the ten countries with the highest age-standardised scabies DALY burdens per 100 000 people were Indonesia 153·86 (95% CI 86·48–254·02), China 138·25 (76·96–225·56), Timor-Leste 136·67 (77·18–221·37), Vanuatu 131·59 (72·56–214·30), Fiji 130·91 (73·01–211·81), Cambodia 126·93 (70·61–214·55), Laos 124·96 (69·32–210·08), Myanmar 124·46 (68·50–208·53), Vietnam 123·30 (68·41–207·56), and Seychelles 122·99 (67·38–203·58; figure 3).

## Discussion

Our analysis of GBD 2015 shows that the greatest burden from scabies is in countries in east Asia, southeast Asia, Oceania, and tropical Latin America. This is supported by previous prevalence studies, which have reported high prevalence of scabies in hot, tropical areas where overcrowding facilitates the rapid spread of the scabies mite.^5^, ^6^, ^7^, ^8^, ^9^ The burden of scabies over the human lifespan has differing patterns in regions with high or low scabies burden. In east and southeast Asia, the regions with greatest scabies burden, DALY burden is highest in children aged 1–4 years, followed by a high but gradually decreasing burden from age 5 to 24 years. DALY burdens decrease substantially during adulthood, before increasing slightly after the age of 70 years. This pattern is much less pronounced in North America and western Europe, which are the regions with the lowest overall scabies burdens. In these low-burden regions, scabies prevalence is more evenly distributed across all age groups, including elderly people, in whom outbreaks of infestations have occurred in care homes.^17^, ^18^

When comparing scabies burden by sex, most world regions had an even distribution between males and females. The greatest discrepancies were in eastern Europe and central Europe, where the ratios of age-standardised DALYs in males to females were 1·23 (eastern Europe) and 0·77 (central Europe). With the exception of North America, age-standardised DALY burdens from 1990 to 2015 across all 21 world regions did not change by more than 8%. North America had the largest mean percent change in age-standardised DALYs from 1990 to 2015, with an increase of 23·8%.

Although beyond the scope of this GBD analysis, subnational differences in the burden of scabies have previously been identified. For example, Aboriginal Australian communities have much higher prevalence of scabies than the non-indigenous population.^19^ Several subnational regions within Ethiopia have been particularly affected by natural disasters such as the El Niño weather phenomenon, leading to severe drought and scabies outbreaks.^20^ Additionally, conflicts in areas such as Africa and the Middle East have led to increasing numbers of refugees seeking asylum in Europe. A recent investigation of a tertiary care hospital in Switzerland found high incidence of co-infection of multiple infectious diseases with scabies in African refugees.^21^

The potential impact of high-quality big data on health and disease is enormous. As the world becomes increasingly globalised, more and more attention is paid to diseases that disproportionately affect vulnerable populations. Although still highly neglected, renewed efforts are now directed toward the global control of scabies. The International Alliance for the Control of Scabies is a global network committed to the control of human scabies and the promotion of health and wellbeing of all those living in affected communities.^22^ The availability of high-quality data on scabies burden, such as those provided by GBD 2015, is needed to enact local, national, and global change.

Funding bodies often consider diseases that disproportionately affect particular populations in their allocation of the limited financial resources. Research interest has recently increased in scabies diagnosis and treatment, and population-based interventions. A particularly notable area that warrants further research and implementation is mass drug administration (MDA) for community-wide control of scabies. A recent comparative study done on several Fijian islands over 12 months showed superior effectiveness of oral ivermectin MDA over topical permethrin in the reduction of scabies prevalence.^23^

The GBD 2015 scabies estimates have some limitations. One notable consideration is the fact that the GBD analysis data on secondary bacterial infection due to *Streptococcus pyogenes* and *Staphylococcus aureus*, which is causally related to the presence of the scabies mite, are not included. Complications of scabies such as impetigo, local and systemic bacterial infections, glomerulonephritis, and rheumatic fever are also not covered. Data from Fiji showed that the attributable risk of scabies infection on impetigo was 94%.^5^ Additionally, the variant of crusted scabies, which has very high mortality, is not considered by GBD. Thus, the GBD assumption that scabies has no mortality (YLL) estimation is entirely focused on the direct skin infection of this condition.

Another crucial deficit is poor case ascertainment in low-resource settings where scabies is most prevalent, including patient presentation, proper disease diagnosis, and adequate coding of scabies. For regions that have missing data, estimates are derived from data in nearby countries, regions, and predictive covariates. As an example, since there are no data sources on scabies epidemiology for sub-Saharan Africa, the estimates for this region are based on global estimates and the covariate of improved water source. The included scabies data sources used in GBD 2015 include US claims reports; however, the USA has a low scabies burden that might have distorted overall global results.

The GBD literature search was done only using PubMed and Google Scholar databases because of time and resource constraints. Thus, regional database searches were not done and the literature search was limited to English and Spanish languages. Since the GBD scabies method does not easily account for outbreaks or particular subgroups (other than age, sex, and geography), certain high-risk groups (eg, homeless populations in the USA) would slightly raise the overall scabies burden estimate for the larger population of which they are a part. The GBD disability weight method is founded on the principle that no disability double-counting occurs. Thus, although the skin effects of scabies can cause more notable morbidity such as sleep deprivation, mental disorders, and renal problems than all other skin conditions, the disability weight for scabies assesses only the direct effect of skin infection and the GBD 2010 Skin Conditions Expert Group assigned it the severity of disfigurement level 1. A final limitation is that GBD modelling does not take into account various diagnostic methods such as different case definitions that might affect diagnosis estimates.

Scabies burden is greatest in tropical regions within east Asia, southeast Asia, Oceania, and tropical Latin America, especially in children, adolescents, and the elderly. Although similar global epidemiological studies are scarce, our findings agree with the systematic reviews and population-level and country-level investigations that we found.^5^, ^6^, ^7^, ^8^, ^9^ GBD 2015 allows for a high-quality, objective measure of scabies burden with regards to its effects on the skin. The premise of GBD is that every human being deserves to live a long life in full health, as described by the IHME. Scabies is an impediment to that goal, particularly in countries with high prevalence and poor access to effective treatment.

Of particular concern is the consistency of the DALY burden from scabies over the past 25 years from 1990 to 2015. In most world regions, no change indicates that scabies burden remains low. However, no change in high-burden regions such as east Asia, southeast Asia, Oceania, and tropical Latin America might indicate inadequate treatment and control measures. Disease treatment and control is particularly difficult, because economically disadvantaged populations are prone to overcrowding and are less likely to afford proper medications or seek appropriate medical attention. Recent studies suggest that MDA campaigns hold the greatest benefit for these regions.
