# Supplementary material for: The global burden of scabies: a cross-sectional analysis from the Global Burden of Disease Study 2015
Source: Lancet Infect Dis. 2017 Dec;17(12):1247–54. doi: 10.1016/S1473-3099(17)30483-8 (PMC5700804; doi:10.1016/S1473-3099(17)30483-8)
Supplement: Supplementary appendix [file mmc1.pdf]

THE LANCET Infectious Diseases

Supplementary webappendix

This webappendix formed part of the original submission and has been peer reviewed. We post it as supplied by the authors.

Supplement to: Karimkhani C, Colombara DV, Drucke AM, et al. The global burden of scabies: a cross-sectional analysis from the Global Burden of Disease Study 2015. *Lancet Infect Dis* 2016; published online September 20. http://dx.doi.org/10.1016/S1473-3099(17)30483-8.

Citation	Country of origin	Year(s) of publication	Details of diagnosis
Paek SY, Koriakos A, Saxton-Daniels S, Pandya AG. Skin diseases in rural Yucatan, Mexico. *Int J Dermatol* 2012; **51**: 823–8.	Yucatan	2009-2010	Dermatologic consultations performed by team of American board-certified dermatologists
Frese T, Herrmann K, Sandholzer H. Pruritus as Reason for Encounter in General Practice. *J Clin Med Res* 2011; **3**: 223–9.	Germany	1999-2000	
Fung WK, Lo KK. Prevalence of skin disease among school children and adolescents in a Student Health Service Center in Hong Kong. *Pediatr Dermatol* 2000; **17**: 440–6.	Hong Kong Special Administration of China	1996-1997	
Perera A, Atukorale DN, Sivayogan S, Ariyaratne VS, Karunaratne LDA. Prevalence of skin diseases in suburban Sri Lanka. *Ceylon Med J* 2000; **45**: 123–8.	Sri Lanka	1997	
Walker SL, Shah M, Hubbard VG, Pradhan HM, Ghimire M. Skin disease is common in rural Nepal: results of a point prevalence study. *Br J Dermatol* 2008; **158**: 334–8.	Nepal	2006	
Popescu R, Popescu CM, Williams HC, Forsea D. The prevalence of skin conditions in Romanian school children. *Br J Dermatol* 1999; **140**: 891–6.	Romania	1995	
Gibbs, S. Skin disease and socioeconomic conditions in rural Africa: Tanzania. *Int J Dermatol* 1996; **35**: 633–9.	Tanzania	1993-1995	
Al-Rubiay KK, Al-Rubaiy LK. Dermatoepidemiology: A Household Survey Among Two Urban Areas In Basrah City, Iraq. *The Internet Journal of Dermatology* 2006; **4**: 10.	Iraq	2005	
Ogunbiyi AO, Omigbodun Y, Owoaje E. Prevalence of skin disorders in school children in southwest Nigeria. *Int J Adolesc Med Health* 2009; **21**: 235–41.	Nigeria	2002-2004, 2006-2008	One dermatologist and three registrars in dermatology unit carried out the physical examinations
Ferié J, Dinkela A, Mbata M, Idindili B, Schmid-Grendelmeier P, Hatz C. Skin disorders among school children in rural Tanzania and an assessment of therapeutic needs. *Trop Doct* 2006; **36**: 219–21.	Tanzania	2003	
Schmeller W, Dzikus A. Skin diseases in children in rural Kenya: long-term results of a dermatology project within the primary health care system. *Br J Dermatol* 2001; **144**: 118–24.	Kenya	1993, 1999	
Oyedeji O, Okeniyi J, Ogunlesi T, Onayemi O, Oyedeji G, Oyelami O. Parental factors influencing the prevalence of skin infections and infestations among Nigerian primary school pupils. *The Internet Journal of Dermatology* 2006; **3**: 6.	Nigeria	2003	
Komba EV, Mgonda YM. The spectrum of dermatological disorders among primary school children in Dar es Salaam. *BMC Public Health* 2010; **10**: 765.	Tanzania	2007-2009	Diagnosis made mainly clinically, laboratory investigations used to confirm difficult diagnoses, specimens obtained from burrows of suspected scabies lesions for direct microcopy of mites
Grover S, Ranyal RK, Bedi MK. A cross section of skin diseases in rural Allahabad. *Indian J Dermatol* 2008; **53**: 179–81.	India	2005	
Abdel-Hafez K, Abdel-Aty MA, Hofny ERM. Prevalence of skin diseases in rural areas of Assiut Governorate, Upper Egypt. *Int J Dermatol* 2003; **42**: 887–92.	Egypt	1994-1996	
Leekassa R, Bizuneh E, Alem A, Fekadu A, Shibre T. Community	Ethiopia	1998	

diagnosis of common skin diseases in the Zay community of the Zeway Islands, Ethiopia. *Ethiop Med J* 2005; **43**: 189–95.			
Chen G-Y, Cheng Y-W, Wang C-Y, Hsu T-J, Hsu MM-L, Yang P-T, Chen W-C. Prevalence of skin diseases among schoolchildren in Magong, Penghu, Taiwan: a community-based clinical survey. *J Formos Med Assoc* 2008; **107**: 21–9.	Taiwan	2005	
Henderson CA. Skin disease in rural Tanzania. *Int J Dermatol* 1996; **35**: 640–2.	Tanzania	1991	
Hay RJ, Castanon RE, Hernandez HA, Lopez GC, Fuentes LF, Solis SP, Andersson N. Wastage of family income on skin disease in Mexico. *BMJ* 1994; **309**: 848.	Mexico	1993	Survey and outpatient clinic visits
Bechelli LM, Haddad N, Pimenta WP, Pagnano PM, Melchior E Jr, Fregnan RC, Zanin LC, Arenas A. Epidemiological survey of skin diseases in schoolchildren living in the Purus Valley (Acre State, Amazonia, Brazil). *Dermatologica* 1981; **163**: 78–93.	Brazil	1974-1975	
Lee J, Koh D, Andijani M, Saw SM, Munoz C, Chia SE, Wong ML, Hong CY, Ong CN. Effluents from a pulp and paper mill: a skin and health survey of children living in upstream and downstream villages. *Occup Environ Med* 2002; **59**: 373–9.	Indonesia	1999	
Ali J, Yifru S, Woldeamanuel Y. Prevalence of tinea capitis and the causative agent among school children in Gondar, North West Ethiopia. *Ethiop Med J* 2011; **47**: 261–9.	Ethiopia	2007-2008	
Woldeamanuel Y, Leekassa R, Chryssanthou E, Menghistu Y, Petrini B. Prevalence of tinea capitis in Ethiopian schoolchildren. *Mycoses* 2005; **48**: 137–41.	Ethiopia	2003-2005	
Mahé A, Prual A, Konaté M, Bobin P. Skin diseases of children in Mali: a public health problem. *Trans R Soc Trop Med Hyg* 1995; **89**: 467–70.	Mali	1993-1994	
Heukelbach J, Wilcke T, Winter B, Feldmeier H. Epidemiology and morbidity of scabies and pediculosis capitis in resource-poor communities in Brazil. *Br J Dermatol* 2005; **153**: 150–6.	Brazil	2001	
Pannell RS, Fleming DM, Cross KW. The incidence of molluscum contagiosum, scabies and lichen planus. *Epidemiol Infect* 2005; **133**: 985–91.	United Kingdom	1994-2003	
Patel JK, Vyas AP, Berman B, Vierra M. Incidence of childhood dermatosis in India. *Skinmed* 2010; **8**: 136–42.	India	2000-2002	Diagnosis made based on a detailed review of history, clinical features, complete physical examination including skin and necessary tests
Libu GK, Bina T, Raphael L, Balakrishnan SE, Biju G, Samson JF, Bindu V. Prevalence and socio-demographic determinants of skin disease among lower primary school children in Calicut, Kerala. *IMAKMJ* 2010; 185–90.	India	2006	
Emodi IJ, Ikefuna AN, Uchendu U, Duru A. Skin diseases among children attending the out patient clinic of the University of Nigeria teaching hospital, *Enug. Afr Health Sci* 2010; **10**.	Nigeria	1996-2005	
Wu YH, Su HY, Hsieh YJ. Survey of infectious skin diseases and skin infestations among primary school students of Taitung County, eastern Taiwan. *J Formos Med Assoc* 2000; **99**: 128–34.	Taiwan	1998	
Dagnew MB, Günther E. Epidemiology of communicable skin diseases in school children of a rural area in North Ethiopia. *Dermatol Monatsschr* 1990; **176**: 219–23.	Ethiopia	1989	
Steer AC, Jenney AWJ, Kado J, Batzloff MR, La Vincente S, Waqatakirewa L, Mulholland EK, Carapetis JR. High burden of	Fiji	2006-2007	Children examined for clinical presence of

impetigo and scabies in a tropical country. *PLoS Negl Trop Dis* 2009; **3**: e467.			impetigo and scabies with the initial examination restricted to the arms legs head and neck to avoid embarrassment, any children with severe scabies or impetigo also had exam of abdomen and back; no exam of buttocks/groin/chest except infants in whom scabies and impetigo lesions are often present in atypical areas, research staff trained in dermatologic clinical examination including the clinical diagnosis of impetigo and scabies and supervised by paediatrician, active impetigo defined as any crusted ulcer or vesiculopustular skin eruption, old impetigo defined as any dry skin lesion, active impetigo classified as mild (less than five lesions) moderate (between five and 20 lesions) or severe (more than 20 lesions), where microbiologic data was available a case of GAS impetigo was defined as a child with one or more active impetigo lesions from which GAS was grown from culture of at least one lesion; Staphylococcus aureus impetigo defined similarly when S. aureus was cultured, scabies defined on basis of typical clinical findings (inflammatory papules with typical distribution that were pruritic) without use of microscopy or dermoscopy, scabies lesions classified according to presence of bacterial superinfection (i.e. scabies or infected scabies).
Kramkimel N, Soussan V, Beauchet A, Duhamel A, Saiag P, Chevallier B, Mahé E. High frequency, diversity and severity of skin diseases in a paediatric emergency department. *J Eur Acad Dermatol Venereol* 2010; **24**: 1468–75.	France	2006	
Ibragimov SI. The structure of skin pathology in middle-aged and	Romania	1989-1990	

elderly subjects. *Vestn Dermatol Venerol* 1990; **2**: 37–40.			
Eason RJ, Tasman-Jones T. Resurgent yaws and other skin diseases in the Western Province of the Solomon Islands. *P N G Med J* 1985; **28**: 247–50.	Solomon Islands	1984	
Harris M, Nako D, Hopkins T, Powell DM, Kenny C, Carroll C, Carroll K. Skin infections in Tanna, Vanuatu in 1989. *P N G Med J* 1992; **35**: 137–43.	Vanuatu	1989	
Feldmeier H, Jackson A, Ariza L, Calheiros CML, Soares V de L, Oliveira FA, Hengge UR, Heukelbach J. The epidemiology of scabies in an impoverished community in rural Brazil: presence and severity of disease are associated with poor living conditions and illiteracy. *J Am Acad Dermatol* 2009; **60**: 436–43.	Brazil	2003	Household leaders asked for room with good light and guaranteed privacy, whole body including the breasts and genital area thoroughly examined for presence of skin lesions typical for scabies, children<10 years only examined in presence of caretaker (mother), diagnosis of scabies made clinically after well-established and approved methods in developing world
Grills N, Grills C, Spelman T, Stoove M, Hellard M, El-Hayek C, Singh R. Prevalence survey of dermatological conditions in mountainous north India. *Int J Dermatol* 2012; **51**: 579–87.	India	2010	Determined by two medical practitioners including trained dermatologist through dermatological history and examination of present family members
Dos Santos MM, Amaral S, Harmen SP, Joseph HM, Fernandes JL, Counahan ML. The prevalence of common skin infections in four districts in Timor-Leste: a cross sectional survey. *BMC Infect Dis* 2010; **61**.	Timor-Leste	2007	Clinical presentation in absence of skin scrapings used for scabies, all unknown conditions were photographed and examined by staff at SPHTMRS for confirmatory diagnosis
Steer AC, Tikoduadua LV, Manalac EM, Colquhoun S, Carapetis JR, Maclennan C. Validation of an Integrated Management of Childhood Illness algorithm for managing common skin conditions in Fiji. *Bull World Health Organ* 2009; **87**: 173–9.	Fiji	2007	Nurse asked parent if their child had skin problem, then proceeded to examine child's skin from head to toe, standard procedure for child to be properly exposed for most aspects of the IMCI guidelines so full skin exam does not cause child further embarrassment
National Center for Health Statistics (NCHS), Centers for Disease Control and Prevention (CDC), United States Census Bureau. United States National Ambulatory Medical Care Survey 1993 and United States National Hospital Ambulatory Medical Care Survey 1993.	United States	1993	
National Center for Health Statistics (NCHS), Centers for Disease Control and Prevention (CDC), United States Census Bureau. United States National Ambulatory Medical Care Survey 1994 and United States National Hospital Ambulatory Medical Care Survey 1994.	United States	1994	

National Center for Health Statistics (NCHS), Centers for Disease Control and Prevention (CDC), United States Census Bureau. United States National Ambulatory Medical Care Survey 1995 and United States National Hospital Ambulatory Medical Care Survey 1995.	United States	1995	
National Center for Health Statistics (NCHS), Centers for Disease Control and Prevention (CDC), United States Census Bureau. United States National Ambulatory Medical Care Survey 1996 and United States National Hospital Ambulatory Medical Care Survey 1996.	United States	1996	
National Center for Health Statistics (NCHS), Centers for Disease Control and Prevention (CDC), United States Census Bureau. United States National Ambulatory Medical Care Survey 1997 and United States National Hospital Ambulatory Medical Care Survey 1997.	United States	1997	
National Center for Health Statistics (NCHS), Centers for Disease Control and Prevention (CDC), United States Census Bureau. United States National Ambulatory Medical Care Survey 1998 and United States National Hospital Ambulatory Medical Care Survey 1998.	United States	1998	
National Center for Health Statistics (NCHS), Centers for Disease Control and Prevention (CDC), United States Census Bureau. United States National Ambulatory Medical Care Survey 1999 and United States National Hospital Ambulatory Medical Care Survey 1999.	United States	1999	
National Center for Health Statistics (NCHS), Centers for Disease Control and Prevention (CDC), United States Census Bureau. United States National Ambulatory Medical Care Survey 2000 and United States National Hospital Ambulatory Medical Care Survey 2000.	United States	2000	
National Center for Health Statistics (NCHS), Centers for Disease Control and Prevention (CDC), United States Census Bureau. United States National Ambulatory Medical Care Survey 2001 and United States National Hospital Ambulatory Medical Care Survey 2001.	United States	2001	
National Center for Health Statistics (NCHS), Centers for Disease Control and Prevention (CDC), United States Census Bureau. United States National Ambulatory Medical Care Survey 2003 and United States National Hospital Ambulatory Medical Care Survey 2003.	United States	2003	
National Center for Health Statistics (NCHS), Centers for Disease Control and Prevention (CDC), United States Census Bureau. United States National Ambulatory Medical Care Survey 2004 and United States National Hospital Ambulatory Medical Care Survey 2004.	United States	2004	
National Center for Health Statistics (NCHS), Centers for Disease Control and Prevention (CDC), United States Census Bureau. United States National Ambulatory Medical Care Survey 2006 and United States National Hospital Ambulatory Medical Care Survey 2006.	United States	2006	
National Center for Health Statistics (NCHS), Centers for Disease Control and Prevention (CDC), United States Census Bureau. United States National Ambulatory Medical Care Survey 2007 and United States National Hospital Ambulatory Medical Care Survey 2007.	United States	2007	

National Center for Health Statistics (NCHS), Centers for Disease Control and Prevention (CDC), United States Census Bureau. United States National Ambulatory Medical Care Survey 2008 and United States National Hospital Ambulatory Medical Care Survey 2008.	United States	2008	
National Center for Health Statistics (NCHS), Centers for Disease Control and Prevention (CDC), United States Census Bureau. United States National Ambulatory Medical Care Survey 2009 and United States National Hospital Ambulatory Medical Care Survey 2009.	United States	2009	
National Center for Health Statistics (NCHS), Centers for Disease Control and Prevention (CDC), SRA International, Inc., United States Census Bureau. United States National Hospital Ambulatory Medical Care Survey 2010 and United States National Ambulatory Medical Care Survey 2010.	United States	2010	
Hogewoning A, Amoah A, Bavinck JNB, Boakye D, Yazdanbakhsh M, Adegnika A, De Smedt S, Fonteyne Y, Willemze R, Lavrijsen A. Skin diseases among schoolchildren in Ghana, Gabon, and Rwanda. *Int J Dermatol* 2013; **52**: 589–600.	Ghana	2007	Physical examination by at least one dermatologist
Clucas DB, Carville KS, Connors C, Currie BJ, Carapetis JR, Andrews RM. Disease burden and health-care clinic attendances for young children in remote aboriginal communities of northern Australia. *Bull World Health Organ* 2008; **86**: 275–81.	Australia	2002-2005	Review of medical records
Lapeere H, Naeyaert J-M, De Weert J, De Maeseneer J, Brochez L. Incidence of scabies in Belgium. *Epidemiol Infect* 2008; **136**: 395–8.	Belgium	2004	Review of medical records
Amro A, Hamarsheh O. Epidemiology of scabies in the West Bank, Palestinian Territories (Occupied). *Int J Infect Dis* 2012; **16**: e117–20.	Palestine	2005-2010	Dermatologist diagnosis
US Hospital Data 2000	United States	2000	
US Hospital Data 2010	United States	2010	
US Hospital Data 2012	United States	2012	
